# Alignment of human KAT2A (GCN5) histone acetyltransferase and SARS‐CoV‐2 Orf8 viral proteins

**DOI:** 10.1002/cdt3.56

**Published:** 2022-12-30

**Authors:** Steven Lehrer, Peter H. Rheinstein

**Affiliations:** ^1^ Division of Pharmacology Fermata Pharma, Inc. New York USA; ^2^ Division of Pharmacology Severn Health Solutions Severna Park Maryland USA

**Keywords:** alignment, protein, virus

The coronavirus SARS‐CoV‐2 has already claimed the lives of more than six million people, according to the World Health Organization, and the actual death toll may exceed 18 million. People are avoiding severe cases of COVID‐19 thanks to vaccines, medications, and immunity from prior infections. Yet the virus's capacity to block the body's immune response, made possible by its arsenal of proteins, is one factor in its propensity to spread.^1^

The SARS‐CoV‐2 virus according to Kee et al. has evolved to mimic KAT2A (lysine acetyltransferase 2A, GCN5), one of the histone proteins that package DNA in the cell nucleus.^2^ Gene transcription is deranged because of this mimicry, which reduces antiviral response. To create chromatin, DNA is wrapped around proteins like histone H3. Among other adjustments, the addition or removal of acetyl groups can modify how tightly chromatin is packed and influence how genes are expressed. An amino acid sequence known as the ARKS motif in H3 is modified by the enzyme KAT2A which adds acetyl groups and encourages gene transcription. Kee et al. found that the Orf8 protein from the SARS‐CoV‐2 virus also has an ARKS motif. KAT2A interacts with Orf8 via ARKS, which modifies it and may cause KAT2A destruction.^3^

Using structures from RCSB Protein Data Bank, we now report another way that Orf8 may interfere with KAT2A and gene transcription. In Orf8, 51 amino acids align closely with KAT2A and could adversely alter its activity.

We examined two RCSB Protein Data Bank molecules: Human GCN5 (KAT2A) histone acetyltransferase (1Z4R)^4^ and SARS‐CoV‐2 Orf8 S84 viral protein (7F5F).^5^

The protein structures were superimposed and aligned on PYMOL (version 2.5.0; Schrödinger, LLC) with the Super command, which super aligns two protein selections. Super does a sequence‐independent structure‐based dynamic programming alignment (unlike the align command) followed by a series of refinement cycles intended to improve the fit by eliminating pairing with high relative variability. The Super command is more reliable than align for proteins with low sequence similarity.

Pymol performed five cycles of calculations on 65 aligned atoms of Human GCN5 histone acetyltransferase and SARS‐CoV‐2 Orf8 S84 viral proteins, with a final root mean square deviation of atomic positions (RMSD) of 0.975 Å for 51 atoms (Figure 1). Lower values of RMSD indicate that alignment is validated with higher accuracy. RMSD values of 1 Å or less indicate very good alignment. The two aligned molecules, Human GCN5 histone acetyltransferase and SARS‐CoV‐2 Orf8 S84 viral protein are shown in Figure 2. The 51‐atom alignment is very good.

**Figure 1:**
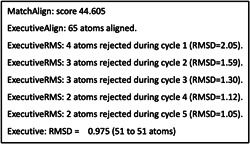
Pymol performed five cycles of calculations on 65 aligned atoms of Human GCN5 histone acetyltransferase and SARS‐CoV‐2 Orf8 S84 viral proteins, with a final root mean square deviation of atomic positions (RMSD) of 0.975 Å for 51 atoms.

**Figure 2:**
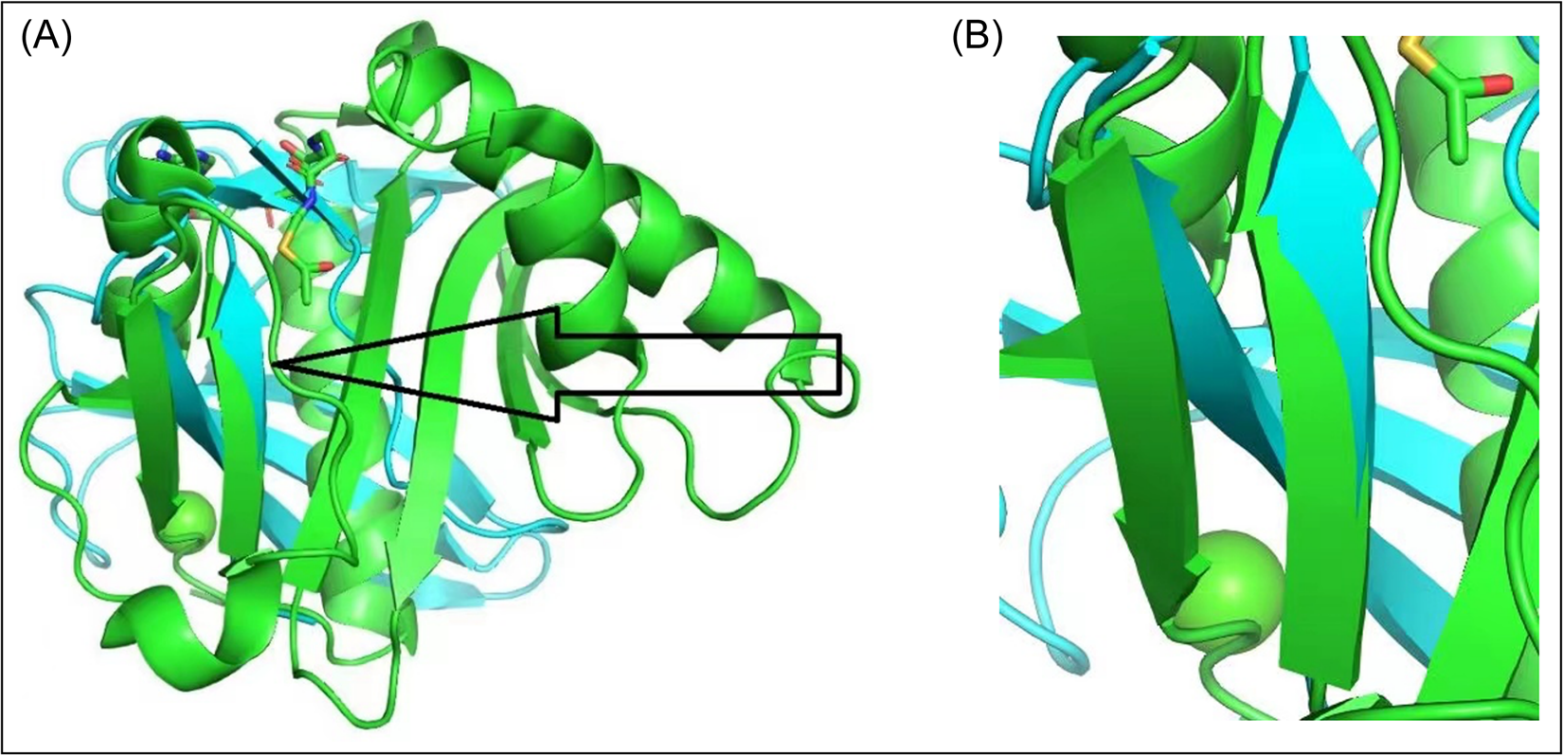
Orf8 aligned with KAT2A (Human GCN5 Acetyltransferase). (A) Black arrow points to closely aligned (RMSD = 0.975 Å) beta sheets of Orf8 (blue) and KAT2A (green). (B) Closeup of aligned beta sheets of Orf8 and KAT2A (center). Green upward pointing arrowhead indicates *TYR 613* of KAT2A; directly underneath is *VAL 117* of Orf8.

A closeup of aligned beta sheets of Orf8 and KAT2A (Figure 2B) indicates *TYR 613* of KAT2A is directly underneath *VAL 117* of Orf8. According to the UCSC Genome Browser, *TYR 613* is in chromosome 17 position 17q21.3 42115759, exon 12. This segment is highly conserved in 100 vertebrates, including the rhesus, mouse, dog, elephant, chicken, western clawed frog, and zebrafish. In addition, the segment is within an H3K27Ac mark often found near regulatory elements (Figure 3).

**Figure 3:**
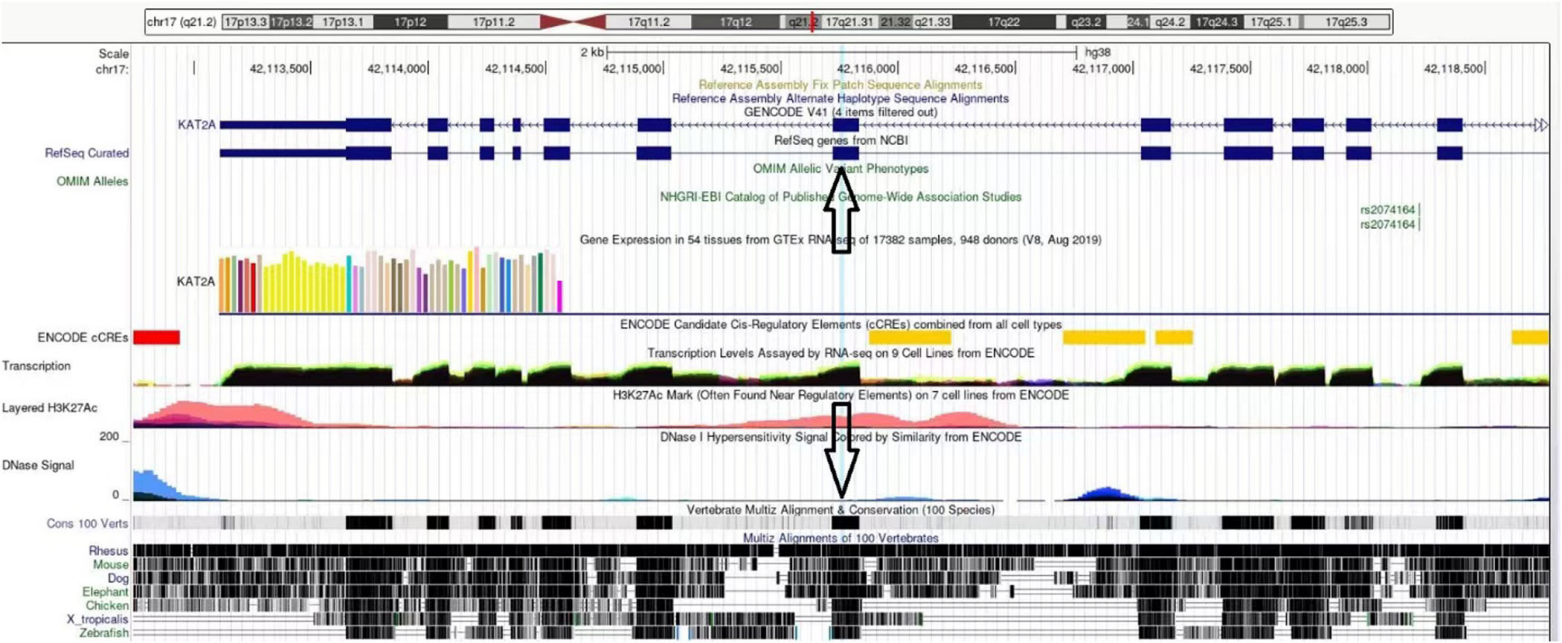
KAT2A in the UCSC Genome Browser. TYR 613 is in chromosome 17 position 17q21.3 42115759, exon 12 (arrows). This segment is highly conserved in 100 vertebrates, including the rhesus, mouse, dog, elephant, chicken, western clawed frog, and zebrafish. In addition, the segment is within an H3K27Ac mark (salmon color under down arrow) often found near regulatory elements.

Figure 4 shows KAT2A with the arrow pointing to the location of alignment with Orf8. Note the open indentation in this position.

**Figure 4:**
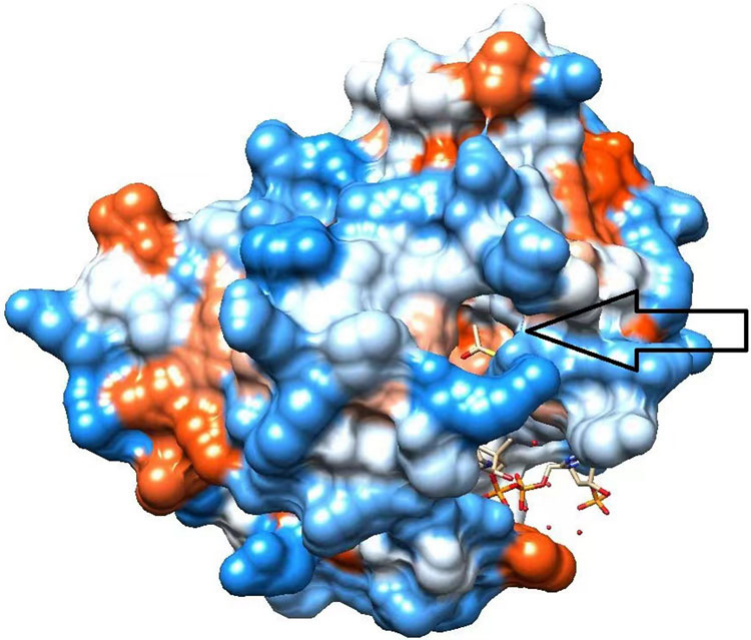
KAT2A with arrow pointing to location of alignment with Orf8. Note the open indentation in this position. Hydrophilic regions are blue, hydrophobic red.

SARS‐CoV‐2 is quite good at blocking host interferon response. Interferons activate hundreds of genes that prevent viral propagation. SARS‐CoV‐2 proteins interfere with interferon response steps, sometimes with multiple proteins inhibiting the same step. For example, SARS‐CoV‐2 Nsp1 binds the ribosomal mRNA channel to inhibit translation.^6^

A protein of 121 amino acid residues and an N‐terminal signal sequence are encoded by Orf8 during SARS‐CoV‐2 infection. Orf8 protein is a dimer that is joined covalently by disulfide links. Orf8 is not necessary for viral replication but does influence how the virus interacts with the host immune system, allowing immune evasion by interfering with KAT2A transcription. KAT2A is a histone acetyltransferase that functions primarily as a transcriptional activator. Orf8 is intensely immunogenic. Persons recovering from SARS‐CoV‐2 infections have high levels of Orf8 antibodies.^7^

The protein structure alignment methods we describe are a powerful way to compare related protein sequences. They can be used to record a variety of information about the matched sequences, such as shared structural function or common evolutionary ancestry. Over the past few decades, protein sequence alignment analyses have become an essential stage in bioinformatics analytic research. Numerous protein databases with information on protein families were created using sequence alignments.^8^

The H3K27ac mark we identified in the 51 amino acid aligned segment of KAT2A suggests that interaction of Orf8 at precisely this spot could disrupt KAT2A transcriptional function. H3K27ac is an epigenetic modification to the DNA packaging protein histone H3. It is an indication that the lysine residue at the histone H3 protein's N‐terminal position 27 has been acetylated. H3K27ac is known as an active enhancer mark because it is connected to greater transcriptional activation. Both the proximal and distal areas of the transcription start site include H3K27ac.^9^

We conclude that the alignment of Human KAT2A Histone Acetyltransferase and SARS‐CoV‐2 Orf8 S84 viral protein we identified suggests a significant effect of Orf8 on KAT2A. Orf8 may interfere with KAT2A gene transcription and disrupt the host cell's ability to regulate gene expression and respond to SARS‐CoV‐2 infection effectively. Since transcription and translation are upregulated in cancer cells, Orf8 could be a cancer treatment.^10^ A small molecule that fits into the open indentation at the site of alignment of Orf8 and KAT2A (Figure 4) might also derange KAT2A gene transcription.

## AUTHOR CONTRIBUTIONS

All authors have accepted responsibility for the entire content of this manuscript and approved its submission.

## CONFLICT OF INTEREST

None.

## ETHICS STATEMENT

None.

## Data Availability

All data publicly available from RCSB Protein Data Bank.
